# Impact of risk adjustment for drug-resistant types on tuberculosis patients’ outcomes under China’s innovative payment methods: a quasi-experimental study design

**DOI:** 10.1186/s40249-026-01423-y

**Published:** 2026-02-15

**Authors:** Mengyuan Zhao, Yingbei Xiong, Yifan Yao, Changli Jia, Kunhe Lin, Li Xiang, Junnan Jiang

**Affiliations:** 1 Department of Health Management, School of Medicine and Health Management, Tongji Medical College, Huazhong University of Science and Technology, Wuhan, China; 2 Department of Labor and Social Security, School of Public Administration, Zhongnan University of Economics and Law, 182 Nanhu Avenue, Wuhan, 430073 China

**Keywords:** Risk adjustment, Tuberculosis, Drug-resistant, Payment reform, China

## Abstract

**Background:**

Treating drug-resistant tuberculosis (DR-TB) is clinically complex and economically burdensome compared to drug-susceptible tuberculosis (DS-TB). China’s diagnosis-intervention packet payment system initially omitted risk adjustment for drug resistance. In 2022, a diagnosis-intervention packet (DIP)-pilot city implemented such adjustment, establishing distinct reimbursement standards for DR-TB and DS-TB. This study aimed to assess the impact of this DR-type risk adjustment on medical expenditures, treatment efficiency, and care quality for TB patients.

**Methods:**

A quasi-experimental difference-in-differences design was employed, involving 8465 TB patients from June 2021 to December 2023. Linear regression was performed with time and treat fixed effects and the interaction term between time and treat. Subgroup analyses for DR-TB and DS-TB patients were conducted.

**Results:**

Under the DIP system, risk adjustment led to marginally significant reductions in inpatient expenditure per hospitalization [*β* = − 151.14, *P* = 0.065; 95% confidence interval (*CI*) for difference in proportions: − 311.66, 9.38] and in annual total inpatient expenditure per patient (*β* = − 200.58, *P* = 0.078, 95% *CI* − 423.26, 22.10) for all TB patients. It also resulted in significant reductions in inpatient out-of-pocket per hospitalization (*β* = − 257.51, *P* < 0.001, 95% *CI* − 316.20, − 198.81), annual total inpatient out-of-pocket per patient (*β* = − 266.78, *P* < 0.001, 95% *CI* − 342.02, − 191.53), inpatient length of stay per hospitalization (*β* = − 3.58, *P* < 0.001, 95% *CI* − 4.53, − 2.62), and annual total length of stay per patient (*β* = − 3.21, *P* < 0.001, 95% *CI* − 4.50, − 1.92). For DR-TB patients, all outcome measures in expenditures, efficiency, or care quality showed *P* > 0.1, indicating no significant changes. For DS-TB patients, measures of expenditures and efficiency showed *P* < 0.1, supporting significant or marginally significant reductions.

**Conclusions:**

The DR-type risk adjustment policy under China’s diagnosis-intervention packet system proved effective in optimizing resource use and enhancing efficiency, particularly for DS-TB patients, while preserving care quality for DR-TB patients. These findings demonstrate the value of tailored risk adjustment within payment frameworks for heterogeneous diseases like tuberculosis, providing crucial evidence for optimizing TB care and implementing effective payment reforms in China and similar settings.

**Supplementary Information:**

The online version contains supplementary material available at 10.1186/s40249-026-01423-y.

## Background

Tuberculosis (TB) has reemerged as the world’s deadliest infectious disease [1], accounted nearly double the mortality burden of HIV/AIDS [2]. Drug-resistant tuberculosis (DR-TB), defined as resistance to at least one first-line anti-TB drug, including monoresistance, polyresistance, multidrug-resistant TB (MDR-TB), and extensively drug-resistant TB (XDR-TB) [3]. DR-TB requires more complex treatment regimens and prolonged treatment than drug-susceptible TB (DS-TB), exacerbating clinical outcomes and socioeconomic inequalities while reducing treatment adherence [4]. Beyond health impacts, DR-TB more likely imposes catastrophic burdens, with treatment costs 3–5 times higher than DS-TB [5, 6], particularly in low-and middle-income countries (LMICs) [7–9]. Notably, in 2023, an estimated 400,000 DR-TB cases were reported globally, with China contributing 7.3% [1]. The substantial burden of DR-TB within China underscores the urgent need for effective interventions [10].

The financial toxicity of TB treatment necessitates payment system innovations [11]. Risk adjustment in payment systems serves a fundamental purpose, which to mitigate perverse incentives in treating high-cost conditions like DR-TB [12, 13]. By stratifying patients based on multidrug resistance status and treatment complexity, these systems align reimbursements with actual resource use—compensating providers fairly for DR-TB while preventing over-treatment of DS-TB [14]. Without such adjustment, providers face financial disincentives to treat DR-TB adequately, potentially compromising care quality and exacerbating health disparities [15, 16].

In October 2020, China launched a major healthcare payment reform, the Diagnosis-Intervention Packet (DIP) system, initially piloted in 71 cities and requiring full implementation across all pilot regions by the end of 2021. The DIP system operates by classifying patients into disease groups using diagnostic codes, assigning cost weights based on historical expenditure, and calculating payments through a global budget pool [17–19]. However, its initial design failed to differentiate DR-TB from DS-TB, creating two distortions: (1) inflated cost-weight allocation for DS-TB cases, and (2) insufficient reimbursement for the complex treatment regimens required by DR-TB [5, 6]. Prior to 2022, the payment standard for each disease group of TB patients was determined by the historical costs of both DR-TB and DS-TB patients. After reform introduced, the payment standards are separated for DR-TB and DS-TB based on their respective historical costs, with the payment standard for DR-TB adjusted upward and for DS-TB adjusted downward. This risk adjustment payment mechanism is expected to optimize medical expenditures by redirecting funds from DS-TB to DR-TB, enhance treatment efficiency through DS-TB payment reductions, and improve care quality via DR-TB reimbursement increases enabling standardized therapies.

Existing literature exhibits two critical gaps. First, though extensive risk adjustment research has been studied in high-income counties [12, 13], evidence remains limited in low-and middle-income countries (LMICs) [20], despite these countries accounting for 99% of global new TB cases [1]. Second, existing evaluations of China’s DIP evaluations have focused on cost control, service efficiency, and healthcare provider behaviors [17–19], while research addressing its risk adjustment mechanisms remains notably limited. Only one study has shown that the absence of a risk adjustment mechanism for DR-TB patients, leading to lower compensation rates, may impede healthcare providers from delivering adequate care [21].

This study adopted a quasi-experimental difference-in-differences (DID) design to assess how risk adjustment for DR type under China’s DIP payment system influences medical expenditures, treatment efficiency, and care quality for TB patients, with explicit stratification between DR-TB and DS-TB cases. By focusing on TB—a disease with significant public health and economic implications—this study provides evidence for precise payment in health insurance, optimizes payment policies, and enhances the efficiency of medical insurance fund use.

## Methods

### Study site

This study was conducted in city A and city B. City A is located in Hubei Province, is a mid-sized city in central China with a population of approximately 4 million. City B is a major economic hub in Guangdong Province with a population exceeding 17 million. Despite differences in economic development and population size, both cities serve as critical DIP pilot regions, ensuring standardized implementation of payment policies. City A launched DIP in June 2021 and implemented DR type risk adjustment in January 2022, whereas city B adopted DIP in March 2020 but didn’t set different payment standards for DR-TB and DS-TB patients through 2024. The differential timing of policy implementation offered a natural experimental framework for this study. It enabled us to harness the policy differences in DIP risk adjustment across cities to analyze the impact of the risk adjustment mechanism on TB patients.

### Study design

This retrospective study evaluated the impact of the DR-type risk adjustment policy on outcomes among TB patients within China’s DIP payment system. The study population comprised patients diagnosed with TB who received care at designated TB treatment centers in city A and city B. These government-designated centers adhere to standardized protocols for TB diagnosis, treatment, and reporting, with centralized care mandated for all DR-TB patients within their respective municipalities.

### Data sources and sample selection

The data for this study were sourced from case records compiled by local authorities, covering the study period from June 2021 to December 2023, the intervention time was January 2022. The data included patients’ demographic information, hospitalization records, and medical expenses. Initially, the specialized hospital for infectious diseases of city A had 3981 records of all hospitalized patients from June 2021 to December 2023, and the specialized hospital for infectious diseases of city B had 19,963 records of all hospitalized patients from January 2018 to June 2024. After screening TB patients with discharge diagnoses of A15 and A16 who were covered by the DIP payment from June 2021 to December 2023, and excluding patients whose treatment period spanned the implementation point of the risk adjustment for DR types, the final sample included 8465 patients for analysis.

To comprehensively evaluate the impact of risk adjustment, the study aggregated annualized patient-level hospitalization data rather than analyzing isolated hospitalization episodes. Prior studies have demonstrated that focusing solely on per hospitalization data may not fully capture the overall impact on patients’ medical expenditures and treatment efficiency [22], particularly for DR-TB, which frequently requires repeated hospital admissions within a single year [7, 23]. The 1-year time frame aligns confounding from longitudinal policy or behavioral changes. Each patient-year observation was treated as a unique unit in our regression models. For patients with multiple admissions within a calendar year, their expenditures, length of stay, and hospitalization counts were summed to create annual totals, and demographic and clinical characteristics were retained as of the first admission in that year. This approach aligns with the study’s aim to assess yearly patient-level outcomes. In all regression models, standard errors were clustered at the patient level to account for within-patient correlation across years. The full process of sample selection is specified in Additional file 1: Fig. S1.

### Variables

The dependent variables spanned three domains: medical expenditures, treatment efficiency, and care quality. Medical expenditure indicators included inpatient expenditure per hospitalization, annual total inpatient expenditure per patient, inpatient out-of-pocket (OOP) expenditure per hospitalization, and annual total inpatient OOP expenditure per patient, which were standardized using the corresponding Consumer Price Index (2021–2023) for medical and health care converted to USD using the annual average exchange rate. Treatment efficiency indicators included inpatient length of stay (LOS) per hospitalization, annual total LOS per patient, and annual total number of hospitalization per patient. The care quality indicator was the 30-day unplanned readmission rate. The key independent variable was the interaction term $Treat*Time$, which captures the policy effect of risk adjustment for DR types. The control variables included per capita disposable income, age, sex, occupation, DR type. Definitions for all the variables are detailed in Additional file 1: Table S1.

### Statistical analyses

We first performed a descriptive statistical analysis of the data. We then employed the DID model, which is a quasi-experimental method that compares changes in outcomes over time between a treatment group and a control group to estimate the causal effect of an intervention, to assess the impact of risk adjustment for DR types on the outcomes of TB patients under DIP payment methods. The model is specified as follows:

$${Y}_{it}={\beta }_{0}+{\beta }_{1}{Treat}_{i}+{\beta }_{2}{Time}_{t}+{\beta }_{3}{(Treat*Time)}_{it}+\gamma {X}_{it}+{\varepsilon }_{it}$$

In this model, ${Y}_{it}$ represents the outcome variables for patient i in year t. ${Treat}_{i}$ is a dummy variable indicating the treatment group assignment, coded as 0 for patients from city B and 1 for patients from city A. ${Time}_{t}$ is a dummy variable indicating whether the observation is before or after the implementation of the risk adjustment policy in January 2022. The key explanatory variable is the interaction term ${(Treat*Time)}_{it}$, which captures the effect of the risk adjustment policy. The coefficient ${\beta }_{3}$ is the core coefficient concerned in this study, which represents the average treatment effect of the risk adjustment on the outcome variables. ${X}_{it}$ is a set of control variables, which are mentioned in the variables sector. ${\varepsilon }_{it}$ is the random error term.

We also performed additional analyses to assess the differential impacts of the risk adjustment on DR-TB and DS-TB patients. Due to the distinct payment standards for DR-TB and DS-TB patients after risk adjustment, we applied the subgroup analysis to determine whether the policy had different effects on two types patients.

Finally, we conducted three types of robustness tests to demonstrate the robustness of the baseline regression results. (1) Parallel trend test. The DID estimators are only meaningful when the parallel trend is satisfied [24], and we plot dynamic treatment effects to indicate the parallel trend. (2) PSM-DID. To address potential selection bias and ensure comparability between the treatment and control groups, we employed propensity score matching (PSM) combined with DID, following prior research [17]. Using logit regression on pre-policy data (June 2021–December 2021), we estimated propensity scores based on control variables and then applied nearest-neighbor matching to select a comparable control group for the treatment group. (3) Placebo test. To assess control group validity, we performed placebo tests by constructing pseudo-treatment groups through random sampling from cases without risk adjustment, matching the actual treatment group size, with remaining cases assigned to pseudo-control groups [25]. This randomized assignment was replicated 500 times to strengthen statistical power. A valid original control group should yield insignificant coefficients near zero, as artificial treatment effects under randomization theoretically lack causal foundations. All statistical analyses were implemented in R (version 4.4.3; R Foundation for Statistical Computing, Vienna, Austria).

## Results

### Descriptive analysis

Between June 2021 and December 2023, a total of 8465 TB patients were included in the analysis, baseline characteristics before and after the risk adjustment implementation are summarized in Table 1. Medical expenditures showed notable reductions in the treatment group after implementation. Inpatient expenditure per hospitalization (from 2350.53 USD to 1822.79 USD) and annual total inpatient expenditure per patient (from 2447.75 USD to 1991.76 USD) decreased substantially. Similarly, both inpatient OOP expenditure per hospitalization (from 1050.43 USD to 785.57 USD) and annual total inpatient OOP expenditure per patient (from 1093.87 USD to 858.39 USD) showed significant decreases in the treatment group. In contrast, changes in the control group for these expenditure metrics were smaller or showed different trends. For treatment efficiency, inpatient LOS per hospitalization decreased significantly in the treatment group post-implementation (from 28.13 to 23.05 days), and annual total LOS per patient also decreased (from 29.30 to 25.19 days). The annual total number of hospitalization per patient remained relatively stable in both groups (treatment: from 1.04 to 1.09; control: from 1.14 to 1.25). For care quality, the 30-day unplanned readmission rate showed minimal change in the treatment group (from 2.70 to 2.89%) but increased in the control group (from 4.91 to 7.35%).

**Table 1:** Baseline characteristics before and after risk-adjustment implementation

Variables	Before implementation		After implementation	
	Treatment	Control	Treatment	Control
Patient characteristics				
Observations, no.	677	1070	2375	4343
Drug resistance type, no. (%)				
DS-TB	600 (88.63)	1064 (99.44)	2216 (93.31)	4249 (97.84)
DR-TB	77 (11.37)	6 (0.56)	159 (6.69)	94 (2.16)
Age, mean (*SD*)	51.97 (18.70)	43.06 (17.02)	57.52 (16.52)	45.42 (18.07)
Sex, number (%)				
Male	407 (60.12)	649 (60.65)	1539 (64.80)	2796 (64.38)
Female	270 (39.88)	421 (39.35)	836 (35.20)	1547 (35.62)
Occupation, number (%)				
Employed	300 (44.31)	652 (60.93)	813 (34.23)	2370 (54.57)
Farmers	107 (15.81)	30 (2.80)	573 (24.13)	220 (5.07)
Retired	73 (10.78)	55 (5.14)	365 (15.37)	494 (11.37)
Unemployed or other	197 (29.10)	333 (31.12)	624 (26.27)	1259 (28.99)
Outcomes				
Inpatient expenditure per hospitalization, mean (*SD*), USD	2350.53 (1808.74)	2191.27 (2053.79)	1822.79 (1099.23)	1902.60 (1602.55)
Annual total inpatient expenditure per patient, mean (*SD*), USD	2447.75 (1901.80)	2515.77 (2436.09)	1991.76 (1376.43)	2378.72 (2324.26)
Inpatient OOP per hospitalization, mean (*SD*), USD	1050.43 (954.19)	460.54 (630.06)	785.57 (523.59)	463.47 (527.11)
Annual total inpatient OOP per patient, mean (*SD*), USD	1093.87 (998.40)	524.79 (703.59)	858.39 (652.68)	584.00 (695.50)
Inpatient LOS per hospitalization, mean (*SD*), day	28.13 (17.14)	10.84 (6.13)	23.05 (11.94)	9.69 (6.42)
Annual total LOS per patient, mean (*SD*), day	29.30 (18.44)	12.40 (7.87)	25.19 (14.47)	12.13 (9.87)
Annual total number of hospitalization per patient, mean (*SD*)	1.04 (0.20)	1.14 (0.44)	1.09 (0.31)	1.25 (0.66)
30-Day unplanned readmission rate, %	2.70	4.91	2.89	7.35

All medical expenditure indicators measured in RMB and converted to USD; Before the risk adjustment implementation: June 2021–December 2021; After the risk adjustment implementation: January 2022–December 2023

*SD*, standard deviation; DS-TB, drug-susceptible tuberculosis; DR-TB, drug-resistant tuberculosis; OOP, out-of-pocket; LOS, length of stay; *CI*, confidence interval; USD: United States Dollar; RMB: Renminbi

### DID estimates of risk-adjustment impact on overall TB patients

Table 2 presents the overall impact of risk adjustment on TB patients. The results indicate that risk adjustment marginally reduced inpatient expenditure per hospitalization by 151.14 USD (95% *CI* − 311.66, 9.38, *P* = 0.065) and annual total inpatient expenditure per patient by 200.58 USD (95% *CI* − 423.26, 22.10, *P* = 0.078). The risk adjustment significantly reduced inpatient OOP per hospitalization by 257.51 USD (95% *CI* − 316.20, − 198.81, *P* < 0.001), annual total inpatient OOP per patient by 266.78 USD (95% *CI* − 342.02, − 191.53, *P* < 0.001), inpatient LOS per hospitalization by 3.58 days (95% *CI* − 4.53, − 2.62, *P* < 0.001), and annual total LOS per patient by 3.21 days (95% *CI* − 4.50, − 1.92, *P* < 0 0.001). No significant impact was observed on annual total number of hospitalization per patient or 30-day unplanned readmission rate.

**Table 2:** DID estimates of risk adjustment on overall TB patient outcomes

Variables	Before implementation		After implementation		DID	95% *CI*		*P*-value
	Treatment	Control	Treatment	Control				
Inpatient expenditure per hospitalization, USD	2350.53	2191.27	1822.79	1902.60	− 151.14	− 311.66	9.38	0.065
Annual total inpatient expenditure per patient, USD	2447.75	2515.77	1991.76	2378.72	− 200.58	− 423.26	22.10	0.078
Inpatient OOP per hospitalization, USD	1050.43	460.54	785.57	463.47	− 257.51	− 316.20	− 198.81	< 0.001
Annual total inpatient OOP per patient, USD	1093.87	524.79	858.39	584.00	− 266.78	− 342.02	− 191.53	< 0.001
Inpatient LOS per hospitalization, day	28.13	10.84	23.05	9.69	− 3.58	− 4.53	− 2.62	< 0.001
Annual total LOS per patient, day	29.30	12.40	25.19	12.13	− 3.21	− 4.50	− 1.92	< 0.001
Annual total number of hospitalization per patient, number	1.04	1.14	1.09	1.25	− 0.04	− 0.10	0.02	0.166
30-Day Unplanned Readmission, %	2.70	4.91	2.89	7.35	0.83	− 0.78	0.42	0.553

All continuous variables are presented as *β* coefficients; Odds Ratios are reported exclusively for 30-day unplanned readmission. All medical expenditure indicators measured in RMB and converted to USD. All regressions controlled individual fixed effects, year fixed effects and covariates. Covariates include average disposable income, gender, age, occupation, and drug-resistant type

DID, difference-in-differences; TB, tuberculosis; OOP, out-of-pocket; LOS, length of stay; *CI*, confidence interval; USD: United States Dollar

### DID estimates stratified by DR-TB and DS-TB subgroups

Tables 3 and 4 present the impacts of the risk adjustment on DR-TB and DS-TB patients. For DR-TB patients, the risk adjustment policy did not have a significant impact on any of the outcome variables. For DS-TB patients, the policy marginally significantly reduced the inpatient expenditure per hospitalization by 146.44 USD (95% *CI* − 309.63, 16.76, *P* = 0.079), significantly reduced annual total inpatient expenditure per patient by 250.75 USD (95% *CI* − 474.06, − 27.45, *P* = 0.028), inpatient OOP per hospitalization by 251.74 USD (95% *CI* − 310.21, − 193.27, *P* < 0.001), annual total inpatient OOP per patient by 290.01 USD (95% *CI* − 362.76, − 217.27, *P* < 0.001), inpatient LOS per hospitalization by 3.55 days (95% *CI* − 4.49, − 2.61, *P* < 0.001), annual total LOS per patient by 3.85 days (95% *CI* − 5.10, − 2.59, *P* < 0.001), annual total number of hospitalization per patient by 0.06 (95% *CI* − 0.12, − 0.01, *P* = 0.031), and no significant impact was observed on 30-day unplanned readmission rate.

**Table 3:** DID estimates of risk adjustment outcomes for DR-TB patients

Variables	Before implementation		After implementation		DID	95% *CI*		*P*-value
	Treatment	Control	Treatment	Control				
Inpatient expenditure per hospitalization, USD	3203.44	3177.77	2529.38	2870.43	− 221.62	− 1826.45	1383.21	0.786
Annual total inpatient expenditure per patient, USD	3582.12	3155.19	3635.45	4325.27	− 1101.27	− 4077.42	1874.88	0.467
Inpatient OOP per hospitalization, USD	1468.98	558.44	1148.23	672.61	− 426.22	− 1102.67	250.23	0.216
Annual total inpatient OOP per patient, USD	1640.45	570.51	1649.05	1029.01	− 507.17	− 1779.27	764.93	0.434
Inpatient LOS per hospitalization, day	32.82	11.00	27.19	10.39	− 4.81	− 16.41	6.79	0.416
Annual total LOS per patient, day	36.19	9.80	38.06	15.49	− 4.02	− 25.00	16.96	0.707
Annual total number of hospitalization per patient, number	1.10	1.00	1.40	1.50	− 0.16	− 0.86	0.54	0.648
30-Day Unplanned Readmission, %	8.05	12.50	10.64	15.98	1.14	− 2.21	2.48	0.910

All continuous variables are presented as *β* coefficients; Odds Ratios are reported exclusively for 30-day unplanned readmission. All medical expenditure indicators measured in RMB and converted to USD. All regressions controlled individual fixed effects, year fixed effects, and covariates. Covariates include average disposable income, gender, age, and occupation

DID, difference-in-differences; DR-TB, drug-resistant tuberculosis; OOP, out-of-pocket; LOS, length of stay; *CI*, confidence interval; USD: United States Dollar; RMB, Renminbi

**Table 4:** DID estimates of risk adjustment outcomes for DS-TB patients

Variables	Before implementation		After implementation		DID	95% *CI*		*P*-value
	Treatment	Control	Treatment	Control				
Inpatient expenditure per hospitalization, USD	2230.46	2184.77	1754.43	1867.54	− 146.44	− 309.63	16.76	0.079
Annual total inpatient expenditure per patient, USD	2302.17	2512.73	1859.94	2332.12	− 250.75	− 474.06	− 27.45	0.028
Inpatient OOP per hospitalization, USD	991.50	459.89	750.48	455.90	− 251.74	− 310.21	− 193.27	< 0.001
Annual total inpatient OOP per patient, USD	1023.73	524.57	794.98	573.34	− 290.01	− 362.76	− 217.27	< 0.001
Inpatient LOS per hospitalization, day	27.47	10.84	22.65	9.67	− 3.55	− 4.49	− 2.61	< 0.001
Annual total LOS per patient, day	28.41	12.42	24.16	12.05	− 3.85	− 5.10	− 2.59	< 0.001
Annual total number of hospitalization per patient, number	1.03	1.14	1.07	1.24	− 0.06	− 0.12	− 0.01	0.031
30-Day Unplanned Readmission, %	1.94	4.86	2.14	7.04	0.82	− 0.92	0.52	0.580

All continuous variables are presented as *β* coefficients; Odds Ratios are reported exclusively for 30-day unplanned readmission. All medical expenditure indicators measured in RMB and converted to USD. All regressions controlled individual fixed effects, year fixed effects, and covariates. Covariates include average disposable income, gender, age, and occupation

DID, difference-in-differences; DS-TB, drug-susceptible tuberculosis; OOP, out-of-pocket; LOS, length of stay; *CI*, confidence interval; USD: United States Dollar; RMB, Renminbi

### Robustness checks

First, to ensure the validity of the DID results, we conducted pre-trend tests using an event study framework, focusing on inpatient expenditure per hospitalization. While the cities differ in size and economic level, no significant differences in pre-policy trends were observed in Additional file 1: Fig. S2. The parallel slopes of the trend lines in the pre-implementation period indicate similar pre-existing trends in the outcome variables. These results suggest that the estimated effects of the risk adjustment policy on TB patients’ outcomes are not due to pre-existing trends, reinforcing the robustness of our findings and supporting the conclusion that the policy had a causal impact on medical expenditures, efficiency, and care quality for TB patients.

Second, the results of the PSM-DID analysis are presented in Additional file 1: Fig. S3 and Tables S2–S4. After matching, the propensity score distributions and kernel density curves demonstrated improved similarity between the groups. The findings were consistent with the original DID estimates, confirming the robustness of our results. For example, the inpatient OOP expenditure per hospitalization showed a significant decrease of 243.19 USD (95% *CI* − 325.20, − 161.18, *P* < 0.001) for all TB patients, and 237.35 USD (95% *CI* − 319.83, − 154.88, *P* < 0.001) for DS-TB patients.

Third, Additional file 1: Fig. S4 displays the distribution of estimates from the 500 placebo iterations with the benchmark estimate. The distribution of estimates derived from random assignments is tightly clustered around zero, indicating no systematic effect from the artificially constructed DR risk adjustment. Furthermore, the empirical *P*-values of all variables are below 0.05, and the benchmark estimates of most variables lie entirely outside their respective placebo distributions. These findings collectively suggest that the observed impact of DR risk adjustment on tuberculosis patients is not attributable to unobserved confounding factors.

## Discussion

Our findings suggest that risk adjustment reimbursement under China’s DIP system may effectively balances quality preservation for DR-TB with cost containment for DS-TB, consistent with the past findings [26, 27]. By establishing a differentiated payment framework, risk adjustment is likely to align the payment standard more consistent with the actual diagnosis and treatment costs and medical resource consumption of patients with different DR types.

For DR-TB patients, the risk adjustment mechanism may have helped maintain care quality without exacerbating patient economic burden. The non-significant downward trends in expenditure and LOS for DR-TB patients may be attributed to two factors. First, the risk adjustment mechanism likely promoted clinical efficiency. By aligning reimbursement with actual treatment costs, the policy reduced financial disincentives for hospitals, enabling greater adherence to standardized protocols and potentially more timely use of effective drug regimens [28]. This may have accelerated recovery, shortened time to discharge, and directly reduced fixed costs. Second, these trends coincided with broader advancements in DR-TB management, such as the adoption of shorter, more potent treatment regimens, which independently contributed to a gradual reduction in treatment duration and cost [1, 2]. The fact that costs did not significantly increase beyond this level supports the conclusion that the new reimbursement was adequate but not excessive, suggesting that external mechanisms like standardized clinical pathways and medical insurance supervision were effective in preventing the financial incentive from leading to resource overuse [7]. For designated tuberculosis-treatment institutions, post-risk adjustment may have aligned payment standards for DR-TB patients with actual medical resource consumption, ensuring adequate reimbursement for healthcare providers and reducing the likelihood of financial losses.

For DS-TB patients, the policy likely contributed to reduced medical expenditures and improved treatment efficiency without compromising care quality. The primary mechanism is likely the recalibration of payment standards downwards, aligning them with actual treatment costs and removing the prior incentive for resource overuse. Prior to risk adjustment, payment standards derived from combined DR-TB and DS-TB historical costs created inflated payment standards for DS-TB cases, encouraging resource overuse. Post-adjustment recalibration aligned DS-TB payments with actual treatment costs, establishing a more precise reimbursement framework. As a result, healthcare providers may have been motivated to optimize treatment processes and reduce unnecessary medical services, as they could no longer rely on loose payment standards to cover potential cost overruns [26]. This is operationally supported by the significant reduction in LOS, indicating streamlined care. Importantly, the concurrent significant decrease in patient OOP expenditures strongly suggests that cost savings were achieved through systemic efficiency gains rather than cost-shifting to patients. While other unobserved hospital-level management initiatives during the study period may have contributed, the temporal alignment with the policy change supports the central role of payment reform.

This study has two main strengths. First, it provides the first evaluation of risk adjustment payment models within China’s innovative DIP framework, specifically among Chinese TB patients. This pioneering evaluation is crucial for understanding the impact of such payment models on medical expenditure, treatment efficiency, and care quality within the specific context of TB treatment in China. Second, this study demonstrates the necessity of risk adjustment payment standards through subgroup analyses for DR-TB and DS-TB patients, highlighting how payment reforms must align with actual treatment costs. For healthcare providers, such adjustments encourage efficient care processes for DS-TB patients while ensuring sufficient resources for DR-TB management, thereby balancing financial viability with clinically appropriate care delivery.

This study has several limitations. First, the risk adjustment policy evaluated in this study applied a broad categorization of DR type. While this represents a crucial initial step towards differentiated payment, this relatively coarse-grained approach may obscure important heterogeneity within the DR-TB patient population. Patients with different resistance profiles (e.g. monoresistance vs. MDR/XDR-TB) likely have significantly varying treatment complexities, resource needs, and costs. Future refinements to the risk adjustment model could explore more granular stratification based on specific resistance patterns or treatment complexity levels to further optimize resource allocation and incentives. Second, DR-TB identification in this study relies strictly on ICD-10 codes, which may pose a risk of undercoding or miscoding, particularly for poly-resistant and XDR-TB categories. Third, unmeasured confounders such as comorbidities, disease severity, and hospital-level factors could influence the results, as only demographic and income variables were included as control variables. Fourth, the relatively small sample size for DR-TB patients may have led to insufficient statistical power, preventing the detection of a true effect. Finally, this study was conducted in two DIP pilot cities with distinct economic and demographic profiles. While the findings provide valuable insights for China’s national DIP reform, regional variations in DR-TB burden and healthcare financing structures may limit direct generalizability. Future multi-center studies across diverse regions are needed to validate the policy’s effectiveness in different settings, particularly in low-resource areas with high DR-TB burdens.

## Conclusions

The risk adjustment policy for DR types has demonstrated its potential in optimizing medical expenditures and enhancing treatment efficiency for TB patients, especially DS-TB patients. The policy's success in DS-TB patients highlights the importance of aligning payment standards with actual medical costs and incentivizing treatment efficiency. For DR-TB patients, the policy did not lead to an increase in inpatient expenditures and OOP payments, and ensured that healthcare providers received reasonable compensation in line with the actual medical resource consumption. These findings provide crucial empirical evidence for China and other countries seeking to optimize TB care and implement effective payment reform strategies.

Based on these findings, we recommend that future DIP payment policies for TB, and potentially other heterogeneous diseases should: (1) sustain and refine risk adjustment based on key factors like DR type, ensuring weights are regularly updated; (2) maintain robust clinical pathways and quality monitoring to safeguard care quality alongside cost efficiency.

## Supplementary Information

Additional file 1. Table S1. Definitions of variables. **Fig. S1**. Sample selection. **Fig. S2**. Tests on the validity of parallel assumptions. **Fig. S3**. Distribution of propensity scores before and after matching. **Table S2**. PSM-DID Estimation of TB Patients’ Outcomes. **Table S3**. PSM-DID estimation of DR-TB patients’ outcomes. **Table S4**. PSM-DID Estimation of DS-TB Patients’ Outcomes. **Fig. S4**. Distribution of estimated coefficients of falsification test.

## Data Availability

The data that support the findings of this study are available from city A Healthcare Security Administration and city B Health Commission but restrictions apply to the availability of these data, which were used under license for the current study, and so are not publicly available. Data are however available from the corresponding author upon reasonable request and with permission of city A Healthcare Security Administration and city B Health Commission.

## Abbreviations

DID: Difference-in-differences
DIP: Diagnosis-intervention packet
DR-TB: Drug-resistant tuberculosis
DS-TB: Drug-susceptible tuberculosis
LMICs: Low- and middle-income countries
LOS: Length of stay
MDR-TB: Multidrug-resistant tuberculosis
OOP: Out-of-pocket
PSM: Propensity score matching
XDR-TB: Extensively drug-resistant tuberculosis
*CI*: Confidence interval
USD: United States Dollar
RMB: Renminbi
