# Supplementary material for: Impact of risk adjustment for drug-resistant types on tuberculosis patients’ outcomes under China’s innovative payment methods: a quasi-experimental study design
Source: Infect Dis Poverty. 2026 Feb 15;15:24. doi: 10.1186/s40249-026-01423-y (PMC12906684; doi:10.1186/s40249-026-01423-y)
Supplement: Supplementary file 1 — Additional file 1. Table S1. Definitions of variables. Fig. S1. Sample selection. Fig. S2. Tests on the validity of parallel assumptions. Fig. S3. Distribution of propensity scores before and after matching. Table S2. PSM-DID Estimation of TB Patients’ Outcomes. Table S3. PSM-DID estimation of DR-TB patients’ outcomes. Table S4. PSM-DID Estimation of DS-TB Patients’ Outcomes. Fig. S4. Distribution of estimated coefficients of falsification test. [file 40249_2026_1423_MOESM1_ESM.docx]

**Supplementary Material**

**A. Sample**

Identifying tuberculosis diagnosis and drug-resistant type.

**B. Exhibits**

**Table s1.** Definitions of variables

**Figure s1.** Sample selection

**Figure s2.** Tests on the validity of parallel assumptions

**Figure s3.** Distribution of propensity scores before and after matching

**Table s2.** PSM-DID Estimation of TB Patients’ Outcomes

**Table s3.** PSM-DID Estimation of DR-TB Patients’ Outcomes

**Table s4.** PSM-DID Estimation of DS-TB Patients’ Outcomes

**Figure s4.** Distribution of estimated coefficients of falsification test

**A. Sample**

**Identifying tuberculosis diagnosis and drug-resistant type.**

In this study, tuberculosis (TB) cases were identified via principal discharge diagnoses under ICD-10 codes A15 (respiratory TB with bacteriological/histological confirmation) and A16 (respiratory TB without bacteriological confirmation). Subsequently, all discharge diagnoses of TB patients were systematically screened for designated drug-resistant (DR) TB codes (ICD-10 extensions indicating monoresistance, polyresistance, multidrug-resistance, or extensive drug-resistance). Patients were classified as drug-resistant tuberculosis (DR-TB) cases if any resistant coding identifiers were present across diagnostic fields. Conversely, those without documented resistance codes in any diagnostic entries were categorized as drug-susceptible tuberculosis (DS-TB) cases.

**Table** ICD-10 Codes for DR-TB cases

| **ICD-10 code** | **Diagnoses** |
| --- | --- |
| A15.000x010 | Secondary pulmonary TB (initial treatment, monoresistant), smear-positive and culture-positive |
| A15.000x011 | Secondary pulmonary TB (initial treatment, monoresistant), smear-negative and culture-positive |
| A15.000x012 | Secondary pulmonary TB (initial treatment, polyresistant), smear-positive and culture-positive |
| A15.000x013 | Secondary pulmonary TB (initial treatment, polyresistant), smear-negative and culture-positive |
| A15.000x014 | Secondary pulmonary TB (initial treatment, extensively drug-resistant), smear-positive and culture-positive |
| A15.000x015 | Secondary pulmonary TB (initial treatment, extensively drug-resistant), smear-negative and culture-positive |
| A15.000x016 | Secondary pulmonary TB (initial treatment, multidrug-resistant), smear-positive and culture-positive |
| A15.000x017 | Secondary pulmonary TB (initial treatment, multidrug-resistant), smear-negative and culture-positive |
| A15.000x020 | Secondary pulmonary TB (retreatment, monoresistant), smear-positive and culture-positive |
| A15.000x021 | Secondary pulmonary TB (retreatment, monoresistant), smear-negative and culture-positive |
| A15.000x022 | Secondary pulmonary TB (retreatment, polyresistant), smear-positive and culture-positive |
| A15.000x023 | Secondary pulmonary TB (retreatment, polyresistant), smear-negative and culture-positive |
| A15.000x024 | Secondary pulmonary TB (retreatment, extensively drug-resistant), smear-positive and culture-positive |
| A15.000x025 | Secondary pulmonary TB (retreatment, extensively drug-resistant), smear-negative and culture-positive |
| A15.000x026 | Secondary pulmonary TB (retreatment, multidrug-resistant), smear-positive and culture-positive |
| A15.000x027 | Secondary pulmonary TB (retreatment, multidrug-resistant), smear-negative and culture-positive |
| A15.100x002 | Secondary pulmonary TB (initial treatment, monoresistant), smear-negative and culture-positive |
| A15.100x005 | Secondary pulmonary TB (initial treatment, multidrug-resistant), smear-negative and culture-positive |
| A15.100x007 | Secondary pulmonary TB (retreatment, monoresistant), smear-negative and culture-positive |
| A15.100x008 | Secondary pulmonary TB (retreatment, polyresistant), smear-negative and culture-positive |
| A15.100x009 | Secondary pulmonary TB (retreatment, extensively drug-resistant), smear-negative and culture-positive |
| A15.100x010 | Secondary pulmonary TB (retreatment, multidrug-resistant), smear-negative and culture-positive |
| A15.500x010 | Bronchial TB (initial treatment, monoresistant), smear-positive and culture-positive |
| A15.500x020 | Bronchial TB (retreatment, monoresistant), smear-positive and culture-positive |

**B. Exhibits**

**Table s1.** Definitions of variables

|  | **Variable name** | **Variable Measurement** |
| --- | --- | --- |
| **Dependent variables** | Inpatient expenditure per hospitalization | Total medical costs per inpatient admission, sourced from detailed hospitalization records |
|  | Annual total inpatient expenditure per patient | Aggregate inpatient costs per patient annually |
|  | Inpatient out-of-pocket (OOP) expenditure per hospitalization | Patient self-paid expenses per admission |
|  | Annual total inpatient OOP expenditure per patient | Cumulative OOP costs per patient annually |
|  | Inpatient length of stay (LOS) per hospitalization | Days spent hospitalized per admission. |
|  | Annual total LOS per patient | Total inpatient days per patient annually |
|  | Annual total number of hospitalizations per patient | Frequency of inpatient visits per year |
|  | 30-day unplanned readmission rate | Percentage of patients readmitted within 30 days of discharge |
| **Independent variable** | Treat | Patients insured by city B local basic medical insurance = 0;  Patients insured by city A local basic medical insurance = 1 |
|  | Time | Before the risk adjustment for drug resistance type implementation = 0;  After the risk adjustment for drug resistance type = 1 |
|  | DID | Treat * Time |
| **Control variables** | Average disposable income | The average income available for consumption and savings for all residents (urban and rural) in a city within a year |
|  | Age | The age of patient |
|  | Sex | Male=1, Female=2 |
|  | Occupation | Employed=1, Farmers=2, Retired=3, Unemployed or other=4 |
|  | Drug-resistant type | Drug-susceptible=0; Drug-resistant=1 |

**
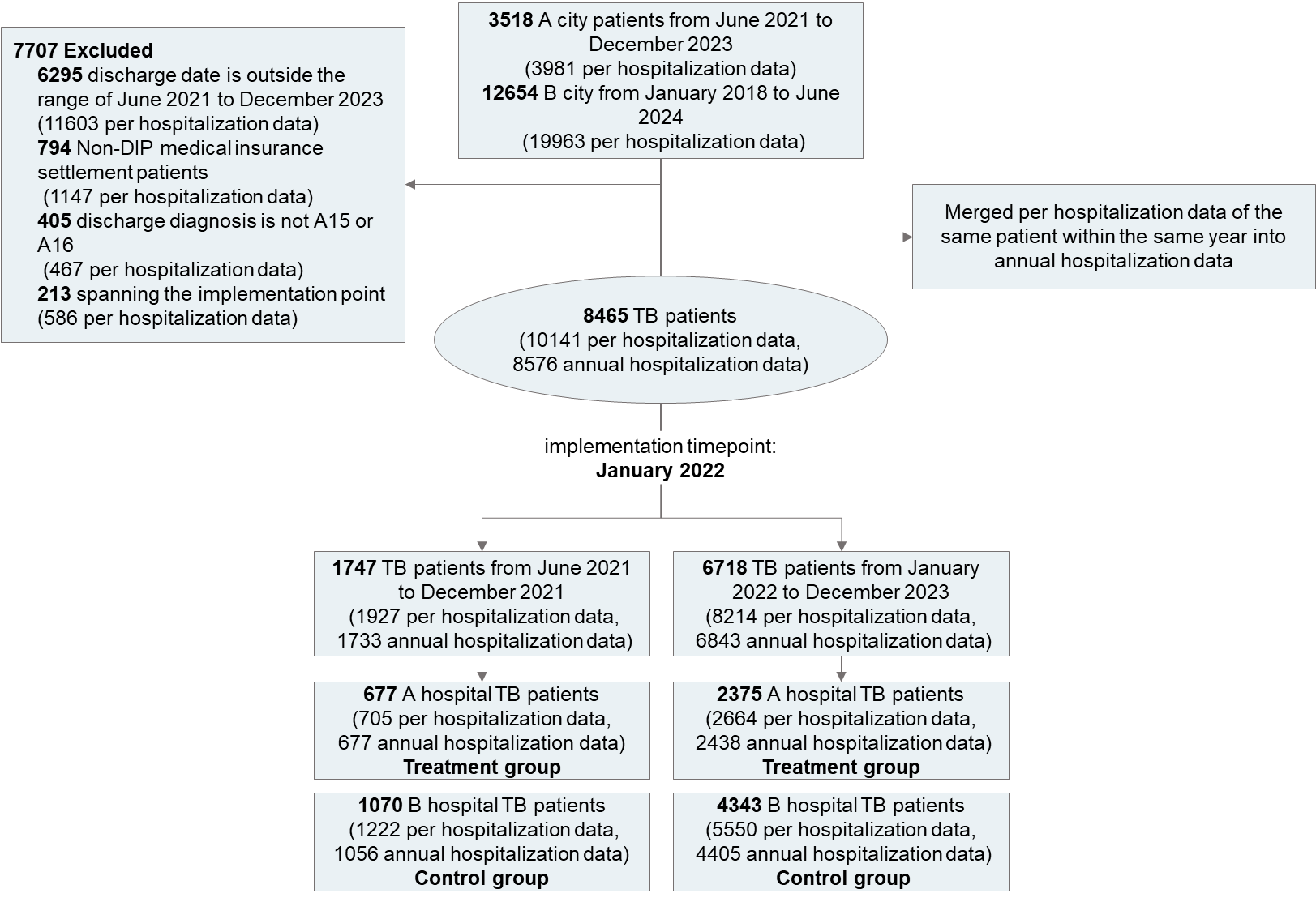
 Figure s1.** Sample selection

**
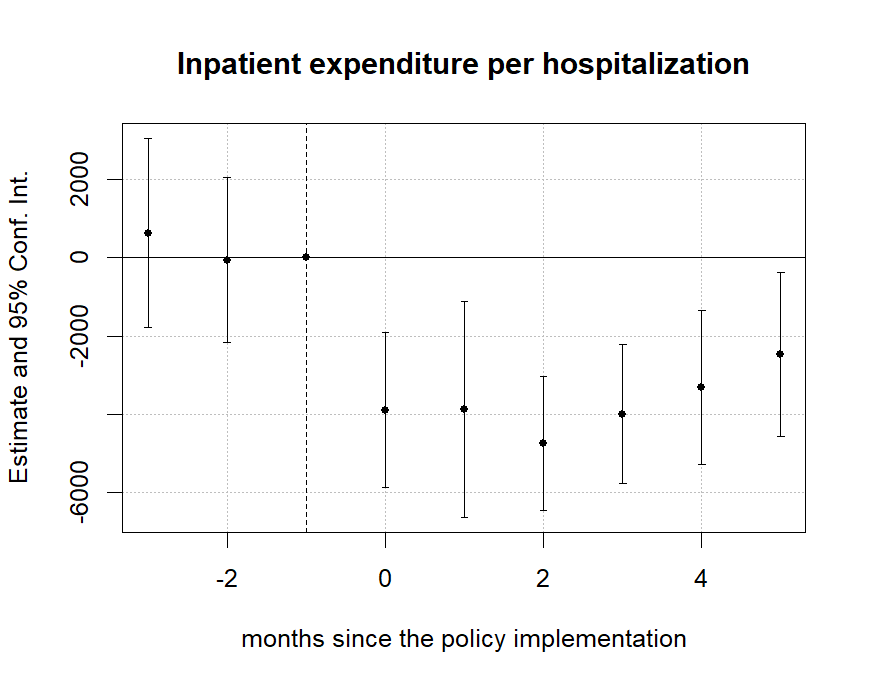
**

**Figure s2.** Tests on the validity of parallel assumptions

Notes: These figures plot the estimated coefficients for interactions between dummies indicating the relative months to the DR type risk adjustment implementation and the treatment group status.


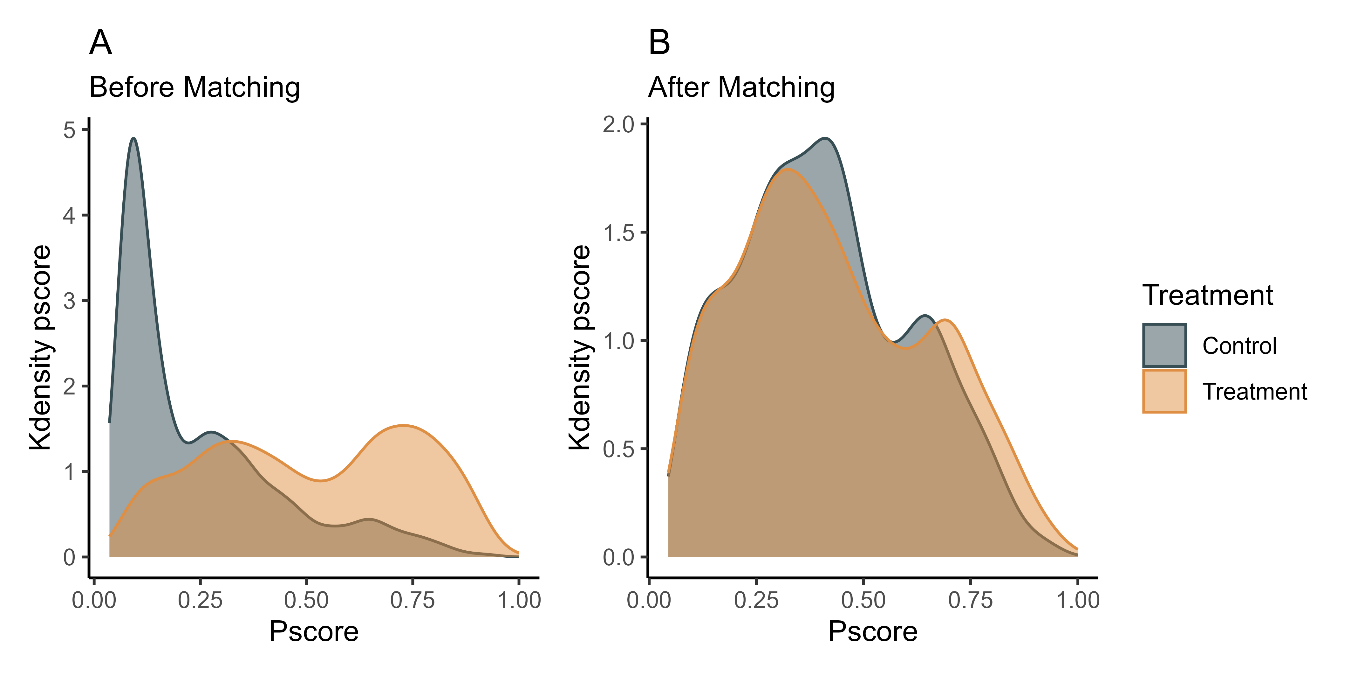


**Figure s3.** Distribution of propensity scores before and after matching

**Table s2.** PSM-DID Estimation of TB Patients’ Outcomes

| Variables | DID | 95% *CI* | | *P*-value |
| --- | --- | --- | --- | --- |
|  |  |  |  |  |
| Inpatient expenditure per hospitalization, USD | 24.78 | -188.94 | 238.50 | 0.820 |
| Annual total inpatient expenditure per patient, USD | -60.81 | -330.53 | 208.91 | 0.659 |
| Inpatient OOP per hospitalization, USD | -243.19 | -325.20 | -161.18 | ＜0.001 |
| Annual total inpatient OOP per patient, USD | -312.10 | -409.02 | -215.18 | ＜0.001 |
| Inpatient LOS per hospitalization, day | -3.75 | -5.13 | -2.36 | ＜0.001 |
| Annual total LOS per patient, day | -2.87 | -4.57 | -1.17 | 0.001 |
| Annual total number of hospitalization per patient, NO. | -0.02 | -0.08 | 0.04 | 0.513 |
| 30-Day Unplanned Readmission, % | 0.80 | -1.03 | 0.58 | 0.592 |

All continuous variables are presented as *β* coefficients; Odds Ratios are reported exclusively for 30-day unplanned readmission. All medical expenditure indicators measured in RMB and converted to USD. All regressions controlled individual fixed effects, year fixed effects and covariates. Covariates include average disposable income, gender, age, occupation, and drug-resistant type.

DID: difference-in-differences; TB: tuberculosis; OOP: Out-of-pocket; LOS: length of stay

**Table s3.** PSM-DID Estimation of DR-TB Patients’ Outcomes

| Variables | DID | 95% *CI* | | *P*-value |
| --- | --- | --- | --- | --- |
|  |  |  |  |  |
| Inpatient expenditure per hospitalization, USD | -30.13 | -1703.12 | 1642.87 | 0.972 |
| Annual total inpatient expenditure per patient, USD | -1024.82 | -4336.59 | 2286.95 | 0.543 |
| Inpatient OOP per hospitalization, USD | -423.86 | -1033.94 | 186.21 | 0.173 |
| Annual total inpatient OOP per patient, USD | -409.34 | -1749.28 | 930.61 | 0.548 |
| Inpatient LOS per hospitalization, day | -5.55 | -16.82 | 5.72 | 0.334 |
| Annual total LOS per patient, day | -1.94 | -23.03 | 19.14 | 0.856 |
| Annual total number of hospitalization per patient, NO. | -0.09 | -0.90 | 0.73 | 0.836 |
| 30-Day Unplanned Readmission, % | 2.67 | -1.59 | 3.55 | 0.454 |

All continuous variables are presented as *β* coefficients; Odds Ratios are reported exclusively for 30-day unplanned readmission. All medical expenditure indicators measured in RMB and converted to USD. All regressions controlled individual fixed effects, year fixed effects, and covariates. Covariates include average disposable income, gender, age, and occupation.

DID: difference-in-differences; DR-TB: drug-resistant tuberculosis; OOP: Out-of-pocket; LOS: length of stay

**Table s4.** PSM-DID Estimation of DS-TB Patients’ Outcomes

| Variables | DID | 95% *CI* | | *P*-value |
| --- | --- | --- | --- | --- |
|  |  |  |  |  |
| Inpatient expenditure per hospitalization, USD | 47.10 | -166.88 | 261.08 | 0.666 |
| Annual total inpatient expenditure per patient, USD | -75.48 | -339.18 | 188.22 | 0.575 |
| Inpatient OOP per hospitalization, USD | -237.35 | -319.83 | -154.88 | ＜0.001 |
| Annual total inpatient OOP per patient, USD | -328.10 | -420.68 | -235.52 | ＜0.001 |
| Inpatient LOS per hospitalization, day | -3.71 | -5.09 | -2.33 | ＜0.001 |
| Annual total LOS per patient, day | -3.34 | -4.99 | -1.69 | ＜0.001 |
| Annual total number of hospitalization per patient, NO. | -0.04 | -0.10 | 0.02 | 0.206 |
| 30-Day Unplanned Readmission, % | 0.66 | -1.30 | 0.47 | 0.356 |

All continuous variables are presented as *β* coefficients; Odds Ratios are reported exclusively for 30-day unplanned readmission. All medical expenditure indicators measured in RMB and converted to USD. All regressions controlled individual fixed effects, year fixed effects, and covariates. Covariates include average disposable income, gender, age, and occupation.

DID: difference-in-differences; DS-TB: drug-susceptible tuberculosis; OOP: Out-of-pocket; LOS: length of stay

**
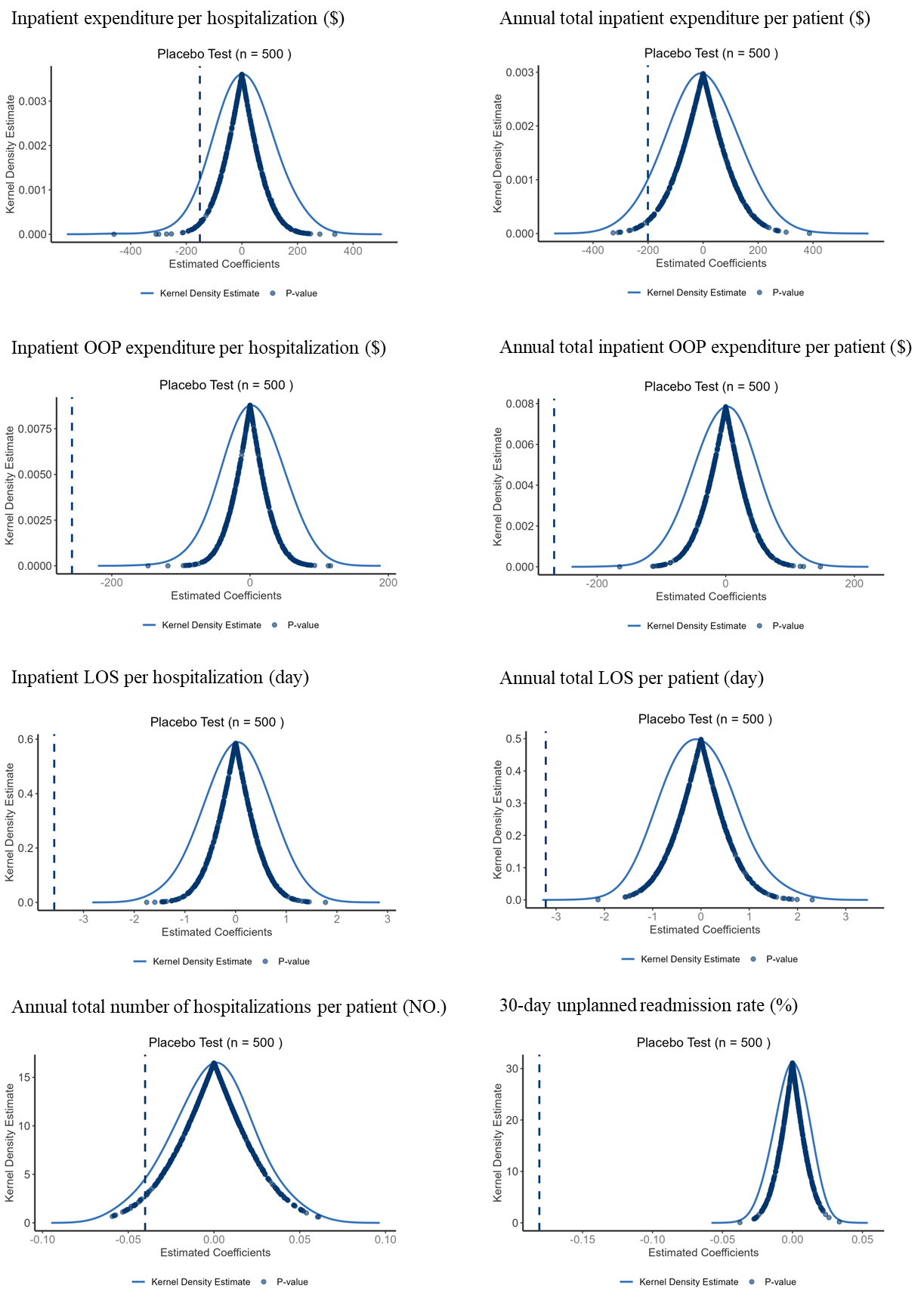
**

**Figure s4.** Distribution of estimated coefficients of falsification test

Note: The figure shows the cumulative distribution density of the estimated coefficients is from 500 simulations randomly assigning the DR risk adjustment to TB patients. The vertical line presents the result of benchmark estimate.
